# Axonal protection by short-term hyperglycemia with involvement of autophagy in TNF-induced optic nerve degeneration

**DOI:** 10.3389/fncel.2015.00425

**Published:** 2015-10-28

**Authors:** Kana Sase, Yasushi Kitaoka, Yasunari Munemasa, Kaori Kojima, Hitoshi Takagi

**Affiliations:** ^1^Department of Ophthalmology, St. Marianna University School of MedicineKawasaki, Japan; ^2^Department of Molecular Neuroscience, St. Marianna University Graduate School of MedicineKawasaki, Japan

**Keywords:** hyperglycemia, autophagy, LC3, p62, tumor necrosis factor, optic nerve

## Abstract

Previous reports showed that short-term hyperglycemia protects optic nerve axons in a rat experimental hypertensive glaucoma model. In this study, we investigated whether short-term hyperglycemia prevents tumor necrosis factor (TNF)-induced optic nerve degeneration in rats and examined the role of autophagy in this axon change process. In phosphate-buffered saline (PBS)-treated rat eyes, no significant difference in axon number between the normoglycemic (NG) and streptozotocin (STZ)-induced hyperglycemic (HG) groups was seen at 2 weeks. Substantial degenerative changes in the axons were noted 2 weeks after intravitreal injection of TNF in the NG group. However, the HG group showed significant protective effects on axons against TNF-induced optic nerve degeneration compared with the NG group. This protective effect was significantly inhibited by 3-methyladenine (3-MA), an autophagy inhibitor. Immunoblot analysis showed that the LC3-II level in the optic nerve was increased in the HG group compared with the NG group. Increased p62 protein levels in the optic nerve after TNF injection was observed in the NG group, and this increase was inhibited in the HG group. Electron microscopy showed that autophagosomes were increased in optic nerve axons in the HG group. Immunohistochemical study showed that LC3 was colocalized with nerve fibers in the retina and optic nerve in both the NG and HG groups. Short-term hyperglycemia protects axons against TNF-induced optic nerve degeneration. This axonal-protective effect may be associated with autophagy machinery.

## Introduction

Glaucoma is a cause of visual field defect due to loss of nerve fibers and thinning of the neuronal rim of the optic nerve head. The risk factors for glaucoma were defined in epidemiological studies (Leske et al., 1994, 2008; Sommer and Tielsch, 1996; Francis et al., 2008; Topouzis et al., 2011). Ocular hypertension, family history, older age, and thinner central corneal thickness are risk factors for primary open-angle glaucoma (POAG; Leske et al., 1994, 2008; Sommer and Tielsch, 1996; Coleman and Miglior, 2008; Francis et al., 2008; Topouzis et al., 2011). It is believed that diabetes mellitus (DM) also influences the pathogenesis and progression of glaucoma (Klein et al., 1994; Leske et al., 1995; Tielsch et al., 1995; Mitchell et al., 1997; Chopra et al., 2008), although the relationship between DM and glaucoma remains controversial. Some recent meta-analyses have shown that DM is risk factor for glaucoma (Zhou et al., 2014; Zhao et al., 2015). It was determined that the pooled relative risk of the association between DM and POAG based on the risk estimate of six cohort studies was 1.40 (Zhou et al., 2014). In contrast, some studies found that glaucoma development is inhibited by DM (Gordon et al., 2002; Akkaya et al., 2014). The Ocular Hypertension Treatment Study showed that among the 191 participants who reported a history of DM at baseline, 6 (3.1%) developed POAG compared with 119 (8.3%) of 1427 participants who did not report a history of DM (Gordon et al., 2002). The univariate hazard ratio for DM was 0.40 and multivariate hazard ratio was 0.37 (Gordon et al., 2002). Moreover, a recent study using Heidelberg retina tomography III has found that rim area and rim volume were significantly higher in POAG patients with DM compared with those without DM and suggested the protective effects of DM against glaucomatous optic nerve damage (Akkaya et al., 2014). Furthermore, a recent double-blind randomized study has shown that 50% glucose eye drops significantly improved the mean contrast sensitivity compared with 0.9% saline in individuals with POAG (Casson et al., 2014). In the animal models, short-term hyperglycemia attenuated axonal degeneration and retinal ganglion cell (RGC) death after experimentally induced ocular hypertension (Ebneter et al., 2011). Although the results of that study suggested that the inhibition of microglial activation is involved in the protective effect of hyperglycemia (Ebneter et al., 2011), its detailed mechanism remains to be elucidated.

Autophagy, or cellular self-digestion, is a cellular pathway involved in protein and organelle degradation which is associated with the pathophysiology of several human diseases (Mizushima et al., 2008). In particular, impaired autophagy has been associated with chronic central nervous system (CNS) diseases (Margulis and Finkbeiner, 2014; Menzies et al., 2015). Recent studies have shown that autophagy is activated by short-term hyperglycemia in Purkinje cells (Yang et al., 2014) and the gastrocnemius (Lv et al., 2014) in rats, and in astrocytes in the brain in mice (Zhang et al., 2010). We have recently demonstrated that the activation of autophagy leads to axonal protection in the hypertensive experimental glaucoma model (Kitaoka et al., 2013) and in the tumor necrosis factor (TNF)-induced optic nerve degeneration model (Kojima et al., 2014). TNF is involved in certain types of glaucoma (Tezel and Wax, 2000; Yan et al., 2000; Yuan and Neufeld, 2000; Tezel et al., 2001; Nakazawa et al., 2006; Sawada et al., 2010), and the TNF injection model may be useful in understanding the mechanism of axonal degeneration of RGCs (Kitaoka et al., 2006; Munemasa and Kitaoka, 2013). Therefore, in the present study, we investigated whether short-term hyperglycemia prevents TNF-induced optic nerve degeneration and examined the role of autophagy in this axon change process.

## Materials and Methods

### Animals

Experiments were performed on 8-week-old male Wistar rats. All studies were conducted according to the Association for Research in Vision and Ophthalmology (ARVO) statement on the Use of Animals in Ophthalmic and Vision Research and were approved by the Ethics Committee of the Institute of Experimental Animals of St. Marianna University Graduate School of Medicine. The animals were housed in controled conditions, with temperature at 23 ± 1°C, humidity at 55 ± 5%, and light from 06:00–18:00.

### Normoglycemic (NG) and Hyperglycemic (HG) Models

The rats were divided into two groups. One group received an intraperitoneal injection of physiological saline solution (PSS) to serve as the normoglycemic (NG) group and the other received an intraperitoneal injection of 60 mg/kg streptozotocin (STZ; Wako, Osaka, Japan) to serve as the HG group. Plasma glucose levels were measured 4 days after intraperitoneal injection using One Touch Ultra View (Johnson & Johnson, Tokyo, Japan). Rats with a plasma glucose level of less than 250 mg/dl in the HG group were excluded from analysis. Plasma glucose levels were also measured 1 and 2 weeks after intravitreal injection.

### Administration of TNF

Both the NG and HG groups received an intravitreal injection of TNF 4 days after intraperitoneal injection of PSS or STZ. Intravitreal injection of TNF was performed as described previously (Kitaoka et al., 2006, 2009). Briefly, rats were anesthetized with an intramuscular injection of a mixture of ketamine-xylazine (10 and 4 mg/kg, respectively). A single 2-μl injection of 10 ng TNF in 0.01 M PBS, pH 7.40, was administered intravitreally to the right eye of an animal under a microscope to avoid lens injury. Phosphate-buffered saline (PBS) alone was administered to the contralateral left eye as a control. In the HG group, 60 mM 3-methyladenine (3-MA; Sigma-Aldrich, St. Louis, MO, USA), an autophagy inhibitor, in dimethyl sulfoxide (DSMO) was administered simultaneously with TNF into the right eyes. The rats were euthanized 1 or 2 weeks after the intravitreal injections with an intraperitoneal overdose of sodium pentobarbital, followed by enucleation of the eyes.

### Immunoblot Analysis

Eighty-four rats were used for immunoblot analysis as described previously (Kitaoka et al., 2011). Briefly, 1 week after intravitreal TNF and PBS injection, optic nerves (4 mm in length starting from immediately behind the globe) were collected, homogenized, and then centrifuged at 15,000×*g* for 15 min at 4°C. Two optic nerve specimens were pooled into one sample. Protein concentrations were determined using the Bio-Rad Protein Assay kit (Bio-Rad, Hercules, CA, USA). Protein samples (2.5 μg per lane) were subjected to SDS-PAGE on gels (Bio-Rad) and transferred to PVDF membranes (Immobilon-P, Millipore, Billerica, MA, USA). Membranes were blocked with Tris-buffered saline (TBS)–0.1% Tween-20 containing 5% skim milk. Membranes were first reacted with anti-microtubule-associated protein light chain 3 (LC3) antibody (1:200; Medical & Biological Laboratories Co., Nagoya, Japan), anti-p62 antibody (1:200; Medical and Biological Laboratories Co.), or anti-β-actin antibody (1:500; Sigma-Aldrich) in TBS containing 5% skim milk. Membranes were then sequentially exposed to peroxidase-labeled anti-rabbit IgG antibody (Cappel, Solon, OH, USA) or peroxidase-labeled anti-mouse IgG antibody (Cappel) diluted 1:5000 in Tween-20 in TBS. Western blots were visualized with an ECL detection system (Amersham ECL Prime Western Blotting Detection Reagents, GE Healthcare, Buckinghamshire, UK).

### Immunohistochemistry

Seven rats in total were used for immunohistochemical study. One week after PBS or TNF injection, optic nerve samples from the NG (3 rats) and HG (4 rats) groups were fixed by immersion in 10% neutral-buffered formalin for 24 h, dehydrated, embedded in paraffin, and sectioned (4 μm thick). Deparaffinized sections were incubated with 1% bovine serum and then reacted with primary antibodies against LC3 (1:200; Medical & Biological Laboratories Co.), or neurofilament-L (a marker of neurons; 1:100; DAKO Corporation, Carpinteria, CA, USA) diluted in 1% bovine serum overnight at 4°C. Sections were then exposed to the following secondary antibodies: FITC-labeled anti-rabbit antibody (1:100; Cappel, MP Biomedicals, LLC) or rhodamine-labeled anti-mouse antibody (1:100; Cappel, MP Biomedicals, LLC). The samples were counterstained with 4′,6-diamidino-2-phenylindole (DAPI; Vesctashield with DAPI, Vector Laboratories, Inc., Burlingame, CA, USA). Negative controls were performed by replacing the primary antibody with PBS or serum.

### Axon Counting

Morphometric analysis of each optic nerve was performed as described previously with samples from 17 rats (Kitaoka et al., 2009, 2011; Kojima et al., 2014). Eyes were obtained from the animals 2 weeks after intravitreal injection. Four-millimeter segments of the optic nerves were obtained starting from 1 mm behind the globe. These segments of optic nerve were fixed by immersion in Karnovsky’s solution for 24 h at 4°C, processed, and embedded in acrylic resin. Cross sections (1 μm thick) were cut beginning 1 mm from the globe and stained with a solution of 1% paraphenylen-diamine (Sigma-Aldrich) in absolute methanol. For each section, images at the center and at each quadrant of the periphery (approximately 141.4 μm from the center) were acquired with a light microscope (BX51; Olympus, Tokyo, Japan) with a 100× coupled digital camera (MP5Mc/OL; Olympus) and associated QCapture Pro software (version 5.1, QImaging, Surrey, Canada). The acquired images were quantified using Aphelion image-processing software (version 3.2, ADISC SA and AAI, Inc., Herouville Saint Clair, France). The number of axons was determined in five distinct areas of 1446.5 μm^2^ each (each quadrant of the periphery in addition to the center; total area of 7232.3 μm^2^ per eye) from each eye. The number of axons per eye was averaged and expressed as the number per square millimeter. A minimum of four eyes per experimental condition was used for analysis.

### Electron Microscopy

Eyes were obtained from animals 2 weeks after intravitreal injection. Four-millimeter segments of the optic nerves were obtained starting from 1 mm behind the globe. These segments of optic nerve were fixed by immersion in Karnovsky’s solution for 24 h at 4°C, processed, and embedded in acrylic resin. Ultrathin sections (approximately 100 nm thick) were prepared using an LKB Ultrotome V (LKB-Produkter AB, Bromma, Sweden), contrasted with saturated aqueous uranyl acetate and Sato’s lead solution, and viewed with a JEOL 1200EX transmission electron microscope (JEOL, Tokyo, Japan) at 80 kV. The number of autophagosomes was determined in 10 distinct areas of 157.3 μm^2^ each (total area of 1573 μm^2^ per eye) from each eye. Three eyes per experimental condition were used for analysis.

### Statistical Analysis

For plasma glucose, data are presented as mean ± S.D. Other data are presented as mean ± S.E.M. Differences among groups were analyzed using Mann-Whitney’s method. A probability value of <0.05 was considered to represent a statistically significant difference.

## Results

### Effects of STZ on Plasma Glucose Levels

There was no significant difference in baseline plasma glucose levels (immediately before intraperitoneal injection, day 0) between the PSS-treated (122.6 ± 6 mg/dl, *n* = 5) and STZ-treated groups (121 ± 6.6 mg/dl, *n* = 6). The plasma glucose levels of STZ-treated HG rats were significantly increased from 4–18 days after intraperitoneal injection of STZ compared with those of PSS-treated NG rats (436.6 ± 59.2 mg/dl and 137 ± 7.5 mg/dl, STZ and PSS groups, respectively, at 4 days, i.e., immediately before intravitreal injection, *n* = 5–6 per group, *p* < 0.01; 477.5 ± 42.1 mg/dl and 172.4 ± 27.6 mg/dl, STZ and PSS groups, respectively, at 11 days, i.e., 1 week after intravitreal injection, *n* = 5–6 per group, *p* < 0.01; 522.1 ± 60.1 mg/dl and 136.6 ± 21.9 mg/dl, STZ and PSS groups, respectively, at 18 days, i.e., 2 weeks after intravitreal injection, *n* = 5–6 per group, *p* < 0.01).

### Effects of Hyperglycemia and 3-MA on TNF-Induced Axonal Degeneration

**Figure 1:**
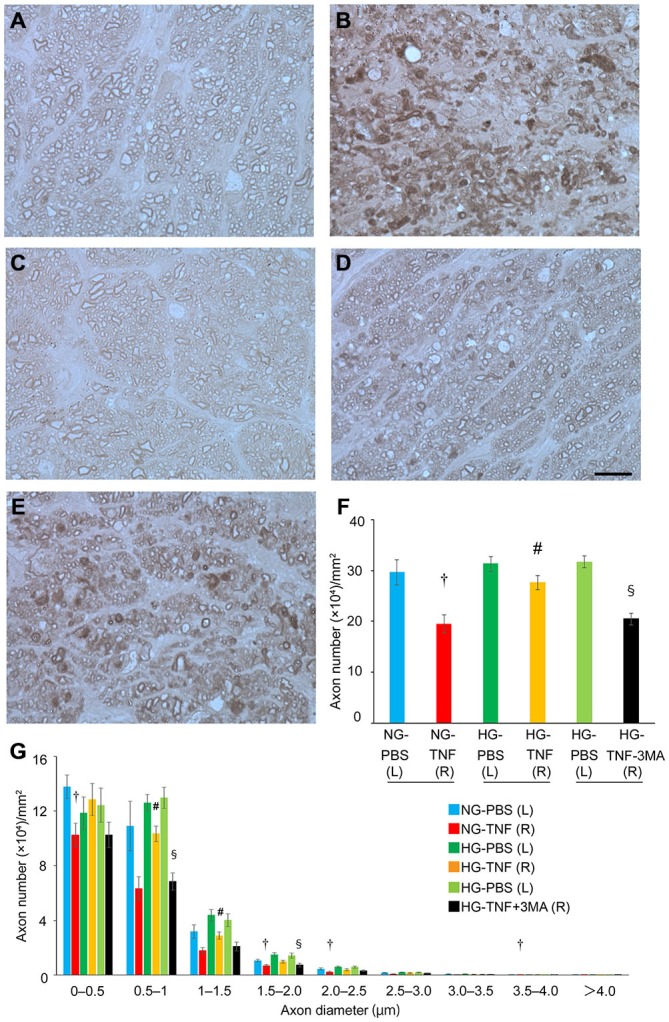
**Short-term hyperglycemia prevented axon loss in TNF-induced optic nerve degeneration.** Light microscopic findings 2 weeks after **(A)** PBS injection with NG, **(B)** 10-ng TNF injection with NG, **(C)** PBS injection with HG, **(D)** 10-ng TNF injection with HG, or **(E)** 10-ng TNF injection ±MA with HG. Scale bar = 10 μm (**A**–**E**). Effect of hyperglycemia, TNF, and 3-MA on axon numbers **(F)** and distribution of axon diameters **(G)** in the optic nerves. Each column represents mean ± S.E.M.; *n* = 4–7 eyes per group. ^†^*p* < 0.05 compared with PBS injection with NG; ^#^*p* < 0.05 compared with TNF injection with NG; ^§^*p* < 0.05 compared with TNF injection with HG. (L), left eyes; (R), right eyes; paired (L) and (R) indicate the same animal.

In PBS-injected eyes, there was no significant difference in axon number between the HG and NG groups (Figures 1A,C,F). Compared with PBS-treated eyes, we confirmed substantial degenerative changes in the axons in the NG group 2 weeks after TNF injection (Figure 1B), which is consistent with the findings of our previous studies (Kitaoka et al., 2006, 2009). The axonal loss after TNF injection was 35% compared with PBS-injected eyes (Figure 1F). However, HG induction exerted a significant protective effect against TNF-induced axonal loss (Figures 1D,F). The HG group showed 84.5% axonal protection compared with the NG group after TNF injection. Similar trends were observed in axon diameter profiles (Figure 1G), although no specific findings were dependent on axon diameter.

Next, we determined the involvement of autophagy in this protective process. Since previous reports showed that autophagy is activated by short-term hyperglycemia (Zhang et al., 2010; Lv et al., 2014; Yang et al., 2014), we examined whether an autophagy inhibitor would affect this axonal protection. As shown in Figure 1E, the protective effect was significantly inhibited by 3-MA, an autophagy inhibitor (Figure 1F). This was also confirmed in axon diameter profiles (Figure 1G).

### Effects of Hyperglycemia, TNF, and 3-MA on LC3-II and p62 Levels in Optic Nerves

**Figure 2:**
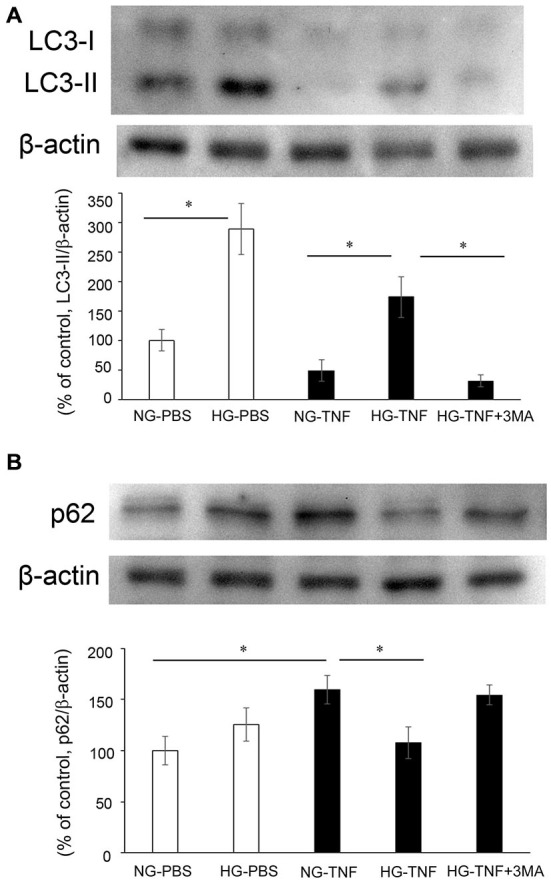
**LC-3 II and p62 protein levels in optic nerves.** Immunoblot data are normalized to β-actin levels in the same sample. All data are expressed as a percentage of control. Each column represents mean ± S.E.M.** (A)** Immunoblotting for LC-3 II 1 week after intravitreal injection. *n* = 5–7 (10–14 eyes) per group. *p < 0.05. **(B)** Immunoblotting for p62 1 week after intravitreal injection. *n* = 6–9 (12–18 eyes) per group. *p < 0.05.

To investigate the effects of hyperglycemia on autophagic status, we examined the changes in LC3-II, an autophagic marker, and p62, a multifunctional protein that interacts with a central component of the autophagy machinery, in the optic nerve. We previously found that axonal loss started 1 week after TNF injection (Kitaoka et al., 2006), and the molecular events before axon loss becomes obvious are important for clarifying the mechanism of axonal degeneration. In PBS- and TNF-injected eyes, there were marked increases in LC3-II protein levels in the optic nerve in HG groups compared with NG groups (Figure 2A). The increase in LC3-II protein levels in HG group was significantly inhibited by 3-MA.

In the NG groups, there was a substantial increase in p62 protein levels in optic nerve samples 1 week after TNF injection (Figure 2B), which is consistent with our previous report in Kojima et al. (2014). In PBS-treated eyes, there was no significant difference in p62 protein levels in the optic nerve between the NG and HG groups (Figure 2B). However, the TNF-induced upregulation of p62 protein levels in the optic nerve in the NG group was significantly inhibited in the HG group (Figure 2B). This inhibitory effect of HG induction was not observed in combination with 3-MA treatment (Figure 2B).

### Effects of Hyperglycemia and TNF on Electron Microscopy Findings

**Figure 3:**
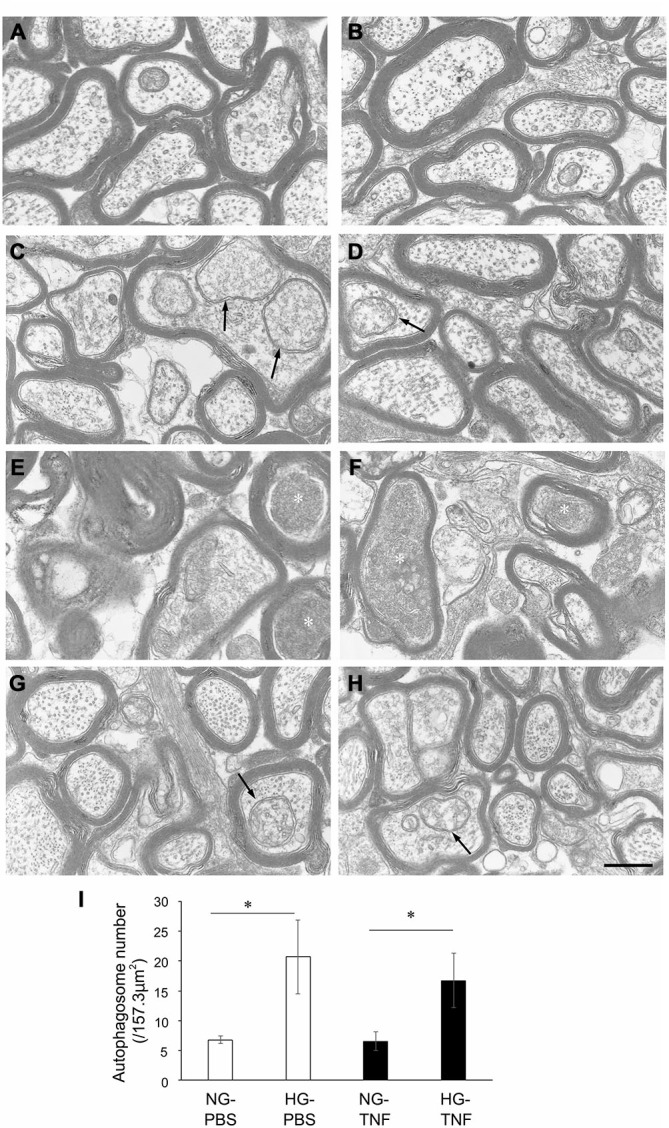
**Autophagosomes can be observed in the optic nerve in short-term hyperglycemia.** Electron microscopy findings 2 weeks after **(A,B)** PBS injection with NG, **(C,D)** PBS injection with HG, **(E,F)** 10-ng TNF injection with NG, and **(G,H)** 10-ng TNF injection with HG. Degenerative changes such as neurofilament accumulation (white asterisks) were noted in the NG-TNF group **(E,F)**. Autophagosomes (black arrows) were observed in the HG-PBS group **(C,D)** and HG-TNF group **(G,H)**. Scale bar = 200 nm. **(I)** Comparison of the numbers of autophagosomes. The number of autophagosomes was determined in 10 distinct areas of 157.3 μm^2^ each (total area of 1573 μm^2^ per eye) from each eye. Each column represents mean ± S.E.M.; *n* = 3 eyes per group. *p < 0.05.

In the NG-PBS group, normal structures of microtubules, neurofilaments, and myelin were observed, but autophagosomes were few (Figures 3A,B). In the HG-PBS group, some autophagosomes were observed inside axons in ɛ the optic nerve and the structures of microtubules, neurofilaments, and myelin were normal (Figures 3C,D). Consistent with our light microscopy findings showing that degenerative changes were apparent, degenerative changes such as neurofilament accumulation and myelin disorganization were observed in the NG-TNF group (Figures 3E,F). However, these degenerative changes were inhibited and well-preserved myelin and microtubule structures were observed in the HG-TNF group (Figures 3G,H). In addition, some autophagosomes were also observed in the HG-TNF group (Figures 3G,H). We counted autophagsome numbers inside axons of the optic nerve. In both PBS- and TNF-injected eyes, there were significant increases in autophagosome numbers in the optic nerves in HG groups compared with NG groups (Figure 3I). These findings are consistent with the substantial increases in LC3-II protein levels seen in the optic nerve in HG groups.

### LC3 in Nerve Fibers in the Retina and Optic Nerve

**Figure 4:**
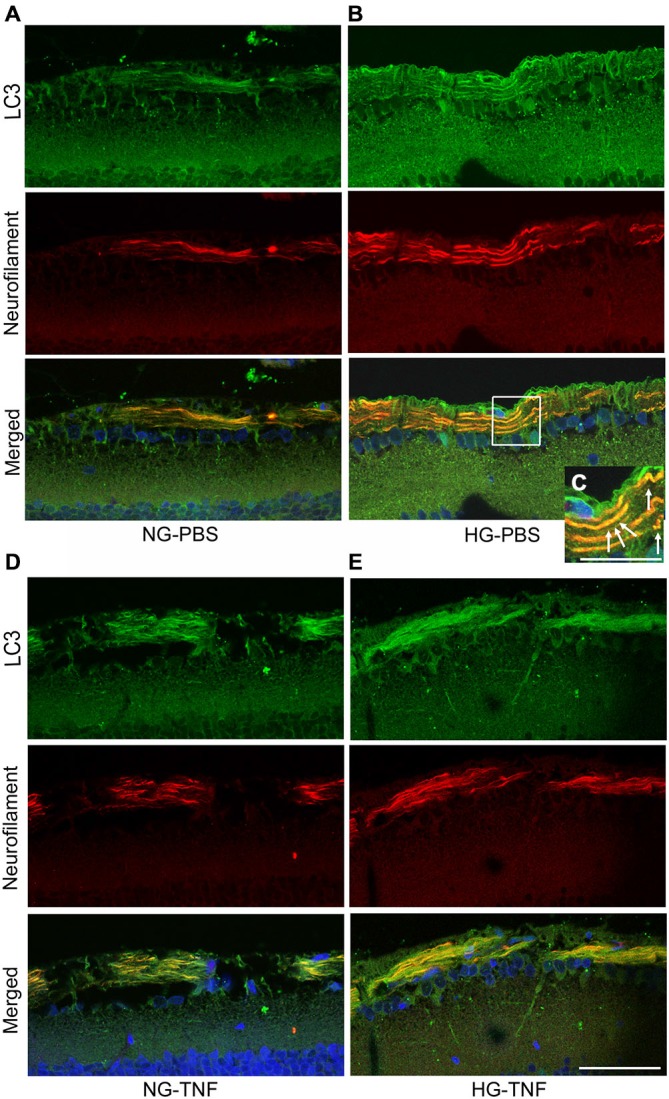
**LC3 is present in nerve fibers in the retina.** Double-staining for LC3 and neurofilaments revealed substantial colocalization in retinal nerve fibers 1 week after PBS injection in the NG **(A)** and HG **(B)** groups, and after TNF injection in the NG **(D)** and HG **(E)** groups. **(C)** High-magnification view of the inset image. Note the LC3-immunopositive dots inside neurofilament-positive fibers (arrows). Scale bar, 50 μm **(A,B,D,E)** and 25 μm **(C)**.

**Figure 5:**
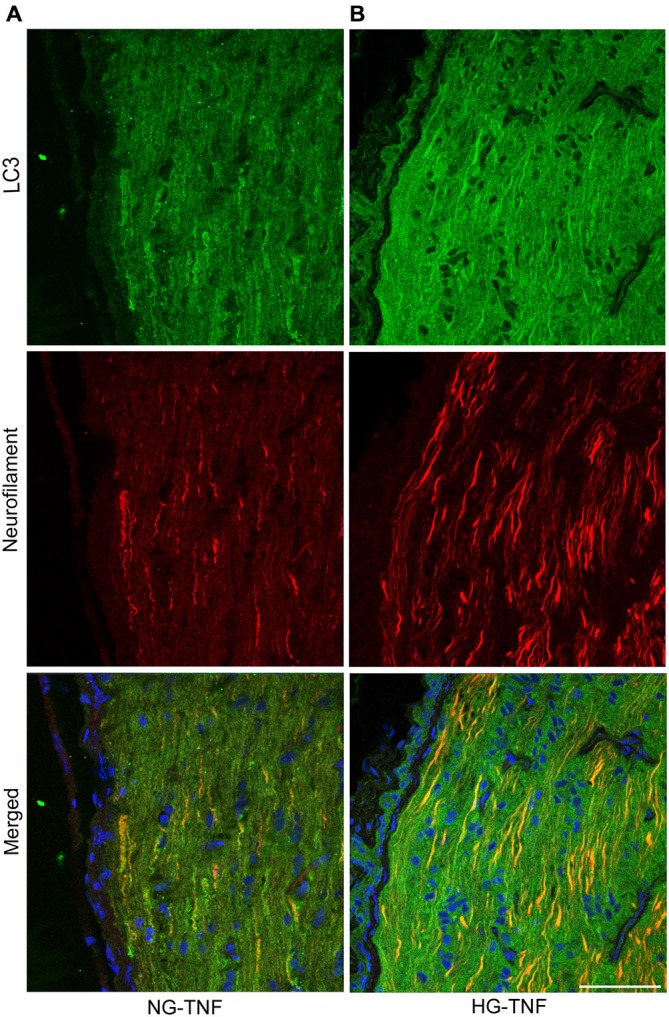
**LC3 is present in the optic nerve.** Double-staining for LC3 and neurofilaments revealed substantial colocalization in the optic nerve 1 week after TNF injection in the NG **(A)** and HG **(B)** groups. Scale bar, 50 μm.

As previously reported in Kim et al. (2008), we observed LC3 immunoreactivity in RGCs in the retina (Figures 4A–E). Double-labeling immunohistochemical studies showed substantial colocalization of LC3 and neurofilaments in the retina in NG and HG rats (Figures 4A–E), suggesting that LC3 is present not only in RGC bodies but also in their axons. Notably, LC3-immunopositive dots were observed inside neurofilament-positive fibers (Figure 4C). In addition, LC3 immunoreactivity appeared to increase in the optic nerves in the HG-TNF group compared with that in the NG-TNF group (Figures 5A,B). Substantial colocalization of LC3 and neurofilaments was also found in the optic nerves (Figures 5A,B).

## Discussion

The present study demonstrated that short-term (2-week) hyperglycemia does not cause axonal degeneration but instead exerts axonal protection against TNF-induced optic nerve degeneration as shown by morphometric analysis of light microscopic as well as by electron microscopic study results. This finding is consistent with previous results showing that short-term hyperglycemia protects retinal neurons including RGCs against ischemic injury (Casson et al., 2004) and protects RGCs and optic nerve axons against ocular hypertension injury (Ebneter et al., 2011). It is also interesting to note that a recent study has demonstrated that glucose rescued cultured rat retinal cells from rotenone-induced toxicity via both the pentose phosphate pathway and the glycolytic pathway, with the maintenance of ATP levels and reduced reactive oxygen species production (Han et al., 2013). On the other hand, we previously found that astrocytes in the optic nerve were activated after TNF injection (Kojima et al., 2012). Therefore, one hypothesis is that TNF enhances glucose utilization by astrocytes and can perturb their energy metabolism (Yu et al., 1995), thereby impairing the ability to provide adequate energy for axons.

The roles of autophagy using rapamycin, an autophagy inducer, and 3-MA, an autophagy inhibitor, on optic nerve axons and RGCs in different injuries remain controversial (Knöferle et al., 2010; Park et al., 2012; Rodríguez-Muela et al., 2012; Deng et al., 2013). However, our present study showed that the protective effect of short-term hyperglycemia was significantly inhibited by 3-MA, suggesting that autophagic machinery mediates this protective effect. Moreover, our recent findings that rapamycin exerts axonal protection against TNF-induced optic nerve degeneration (Kojima et al., 2014) also support the current findings. In addition, it is noteworthy that high glucose-induced autophagy protects bone marrow-derived mesenchymal stem cells from apoptosis (Chang et al., 2015).

The amount of LC3-II correlates closely with autophagosome number (Mizushima et al., 2010). Our immunoblot analysis showed substantial increases in LC3-II protein levels in the optic nerve in HG groups compared with NG groups. Consistent with this, the present electron microscopic study showed that noticeable autophagosomes were observed in both the HG-PBS and HG-TNF groups and quantification confirmed that their number increased. STZ is an antibiotic that can cause pancreatic β-cell destruction (Wu and Huan, 2008), thereby leading to low levels of plasma insulin. A previous report showed that under HG conditions induced by glucose infusion, autophagy was inhibited in rat skeletal muscle, whereas under STZ-induced HG conditions, autophagy was enhanced (Lv et al., 2014). This difference in autophagic status suggests that insulin may regulate autophagy machinery (Lv et al., 2014). During hyperglycemia, the administration of insulin reduced LC3-II levels in type 2 diabetes patient muscle (Kruse et al., 2015), implying that low levels of insulin may lead to increased LC3-II levels. Taken together, the results suggest that the increases in LC3-II protein levels in the optic nerve may be associated with autophagy activation in STZ-induced HG rats that have low levels of insulin.

We observed LC3-immunopositive dots inside neurofilament-positive fibers near RGC bodies and in the optic nerve. Since LC3 is known to occur on autophagosomes and widely used as a marker of autophagosomes (Kabeya et al., 2000; Mizushima et al., 2010), the presence of LC3 in nerve fibers is consistent with our electron microscopy findings indicating the presence of autophagosomes inside axons. It is particularly important to note that autophagosome biogenesis occurs in distal neurites and then moves mainly retrogradely to cell soma in dorsal root ganglia neurons (Maday et al., 2012). Although it is not clear whether this is the case for optic nerve axons and RGC soma, it is possible that increased LC3 protein levels in the optic nerve resulted in abundant LC3-immunopositive dots in all neurofilament-positive fibers in the HG group.

A previous report showed that hyperglycemia does not affect p62 protein levels in human muscle (Kruse et al., 2015). However, in the present study, the short-term hyperglycemia significantly inhibited TNF-induced upregulation of p62 protein levels in the optic nerve. Consistent with this finding, a previous study demonstrated that the p62 protein level in brain tissue was significantly decreased in STZ-induced hyperglycemia compared with normoglycemia in a cerebral ischemic mouse model (Wei et al., 2013). Since decreased p62 levels indicate enhanced autophagic flux (Bjørkøy et al., 2009), one possibility is that STZ-induced hyperglycemia may lead to increased autophagic flux in the optic nerve. Because we observed a difference in the p62 level between the NG and HG groups only in TNF-injected eyes, hyperglycemia may improve autophagic flux in TNF-induced optic nerve degeneration, although this may not occur in PBS-injected eyes. It is proposed that enhanced autophagic flux leads to increasing clearance of unnecessary proteins and therefore may be beneficial for neuroprotective interventions in optic neuropathy (Munemasa and Kitaoka, 2015). We only observed axonal protection over the short term and expect that long-term hyperglycemia will lead to neurodegeneration, as shown in other neuronal cells that exhibited autophagic activity impairment and degeneration in the Purkinje neurons of 24-week STZ-HG rats (Yang et al., 2014).

In conclusion, our present results suggest that short-term hyperglycemia protects axons in TNF-induced optic nerve degeneration and that this axonal-protective effect may be associated with autophagy machinery.

## Author Contributions

KS, YK, YM and KK performed experiments; KS, YK and HT designed the research protocol and analyzed data; and KS, YK and KK wrote and YM and HT revised the article. All authors have read and approved the final manuscript.

## Conflict of Interest Statement

The authors declare that the research was conducted in the absence of any commercial or financial relationships that could be construed as a potential conflict of interest.

## Abbreviations

NG: normoglycemic
HG: hyperglycemic
STZ: streptozotocin
LC3: microtubule-associated protein light chain 3
POAG: primary open-angle glaucoma
RGC: retinal ganglion cell
TNF: tumor necrosis factor
PBS: phosphate-buffered saline
3-MA: 3-methyladenine
DAPI: 4′,6-diamidino-2-phenylindole
DMSO: dimethyl sulfoxide
TBS: Tris-buffered saline
PSS: physiological saline solution.
